# Prognostic value of number of negative lymph node in patients with stage II and IIIa non-small cell lung cancer

**DOI:** 10.18632/oncotarget.18154

**Published:** 2017-05-24

**Authors:** Shengguang Wang, Bin Zhang, Chenguang Li, Chao Cui, Dongsheng Yue, Bowen Shi, Qiang Zhang, Zhenfa Zhang, Xi Zhang, Changli Wang

**Affiliations:** ^1^ Department of Lung Cancer, Tianjin Medical University Cancer Institute and Hospital, Tianjin, 300060, China; ^2^ Tianjin Lung Cancer Center, Tianjin, 300060, China; ^3^ Tianjin Key Laboratory of Cancer Prevention and Therapy, Tianjin, 300060, China; ^4^ National Clinical Research Center for Cancer, Tianjin, 300060, China; ^5^ Graduate School, Tianjin Medical University, Tianjin, 300070, China; ^6^ Department of Thoracic Surgery, Tianjin Haihe Hospital, Tianjin, 300350, China; ^7^ Affiliated Yueqing Hospital, Wenzhou Medical University, Wenzhou, Zhejiang, 325000, China; ^8^ School of Pharmaceutical Sciences, Wenzhou Medical University, Wenzhou, Zhejiang, 325000, China

**Keywords:** NLN, RML, NSCLC, prognostic factor

## Abstract

**Background:**

The definitive validation evidence of the implications of lymph node metastases regarding the survival of Non-Small Cell Lung Cancer (NSCLC) patients is lacking. We aimed to evaluate the prognostic impact of several lymph node metastases-associated risk factors including Number of Negative Lymph Node (NLN) and risk-stratify NSCLC patients into subsets with different prognosis.

**Method:**

A total of 482 patients with N1 and N2 NSCLC were included in this study. The prognostic importance of a set of risk factors was examined by univariate and multivariate analysis. The cut-off points and 5 years survival rates were calculated to test the best grouping system to stratify the patients with difference outcome.

**Results:**

Our analysis indicated that both Ratio of the Metastatic Lymph nodes (RML) and Number of Negative Lymph Node (NLN) were associated with overall survival (OS) and disease free survival (DFS). RML percentage 20% and 55%, and NLN counts 10 and 30 were proved as the optimal cut-off points to predict OS by classifying patients into 3 groups, respectively. RML and NLN actually are more powerful in predicting survival outcome for male patients compared to female patients. Stratified survival analyses using combined factors indicated that the 5-year survival rate (5-YSR) is high in RML I + NLN I/III subgroup (5-YSR = 57.1% and 43.3%) and low in RML III + NLN II/III subgroup (5-YSR = 0.0 % each).

**Conclusions:**

NLN is a strong prognostic factor for OS and DFS of stage II/IIIa NSCLC patients, and provides a useful classification scheme for NSCLC patients when combined with RML.

## INTRODUCTION

Non-Small Cell Lung Cancer (NSCLC), accounting for approximately 85% of all lung cancers, has become a leading cause of cancer-related mortality all around the world [1]. The current TNM classification system of the American Joint Committee on Cancer defines N status (Nx, N0, N1, and N3) depending on the localized spread to the regional lymph nodes of NSCLC. Previous studies in node-negative NSCLC patients have consistently reported the survival of Nx (no lymph node examined) and N0 (lymph node negative) NSCLC patients are significantly different [2-4]. However, the survival status, disease outcome and choice of treatment for node-positive NSCLC patients is controversial [5].

In clinical practice, many other markers have been tested for their predictive and prognostic value in NSCLC, as dependent or independent factors of TNM classification. The presence of lymph node metastases has displayed more important implications regarding prognosis, recurrence and overall survival for cancer patients [6-8]. Ratio of the Metastatic Lymph nodes (RML) is the ratio between metastatic and examined lymph nodes which sometimes is termed as Lymph Node Ratio (LNR). It is suggested RML (or LNR) provided independent prognostic data in NSCLC in all N stages [9-11].

Number of Negative Lymph Node (NLN) recently emerged as a new prognostic factor for node-positive cancer. NLN improved the prognostic prediction of TNM classification in various types of cancer [12-15]. For instance, NLN was found to improve the prognostic prediction for gastric cancer and breast cancer patients [16-18]. Combining the NLN with RML better predict the postoperative survival in cervical cancer patients [19]. The prognostic value of NLN in NSCLC patients remains unclear.

In this study we identified the optimal cut-off points of NLN in node-positive NSCLC patients (mostly in stage IIIa) and our analyses showed that NLN is associated with significant prognostic value in survival prediction. We also stratified distinct subsets of patients in respect to the combined use of NLN and RML. Our data suggested NLN, together with the RML, could develop a useful classification scheme for NSCLC patients to predict outcome of one’s chance of survival.

## PATIENTS AND METHODS

### Patients

A total of 482 patients with locally advanced NSCLC at the Tianjin Medical University Cancer Institute and Hospital were included in this study between January 12, 2004 and January 18, 2011. Criteria for inclusion in this study included: 1) Primary NSCLC histologically confirmed in stage II or IIIa according to new 7^th^ edition TNM classification at Tianjin Medical University Cancer Institute and Hospital; (2) Received complete resection of the tumor; (3) More than 10 lymph node dissection. Patients received chemotherapy, section surgery or died perioperative period were excluded. All patients were followed up every 3 months for the first 2 years, every 6 months during the following 3 years and once a year thereafter. In this study, every patient received at least 5 years follow-up or until death.

This retrospective study was approved by the Research Ethics Committee of the Tianjin Medical University Cancer Institute and Hospital. Written documentations of informed consent were provided by all patients.

### Data collection

Clinical and pathological data were obtained from clinical records and summarized in Table 1. The data included but not limited to gender, age, smoking history, type of pulmonary lobectomy, primary tumor location, TNM classification, tumor staging, type of surgery, and recurrence status. Overall survival (OS) and disease free survival (DFS) data were collected during follow up for each patient.

**Table 1:** Clinical and demographic characteristics of the patients (*N*=482)

Characteristics	*N*	%
Gender, Male	301	62.4
Age at surgery, <65y	150	31.1
Smoking, Yes	302	62.7
Type of Pulmonary Lobectomy, Subtotal/Lobe/Sleeve	364/111/7	75.5/23.0/1.5
Location of Primary Tumor, Peripheral/Central	310/172	64.3/35.7
Subcarinal Lymph Node, Positive	191	39.6
T Stage (T1/T2/T3)	151/271/52	32.6/56.2/10.8
N Stage (N1/N2)	107/375	22.2/77.8
Pathological Stage (IIa/IIb/IIIa)	77/23/382	16.0/4.8/79.3
Postoperative recurrence, Yes	377	78.2
Adjuvant chemotherapy, Yes	346	71.8

### Statistical analysis

All analyses were performed using the SPSS version 21.0 software (IBM, Chicago, IL, USA) with 2-sided p values. All categorical variables were analyzed by standard descriptive statistics and presented as numbers and percentages. The cut-point survival analyses were performed as described previously in other type of cancer [20]. The patients were divided into three groups based on the cut-offs of RML or NLN. RML was calculated as the ratio between metastatic (positive) lymph node number and examined lymph node number. NLN was calculated as the total lymph nodes number minus positive lymph nodes number. The univariate analysis and the survival curves of both OS and DFS were created using the Kaplan-Meier method and the survival difference were compared using the log-rank test. Multivariate analyses of OS were conducted using Cox’s proportional hazard model (ENTER method) to identify independent prognostic factors. The bivariate correlation analysis was performed to evaluate the correlation between NLN and other variables. The Pearson’s correlation coefficient was provided to measure the strength and direction of linear relationships. Statistical significance was defined as a *p* value < 0.05 (*) or < 0.01 (**).

## RESULTS

### Patient information

The overall demographic and clinical characteristics of the study subjects were summarized in Table 1. Over the study period, a total of 482 NSCLC patients (62.4% male) who met the inclusion criteria were enrolled in this study. Clinical stages IIa, IIb and IIIa were found in 16.0%, 4.8%, and 79.3%, respectively. 31.1% (150) of the patients are younger than 65-year old; 62.7% (302) had smoking history; 75.5% (364) had lobectomy. Of the patients whose tumor information was available, 364 (75.5%) tumors were located in peripheral. The TNM classification was applied for staging purposes. Overall, 32.6% (151) and 56.2% (271) patients were staged as T1 and T2, while 22.2% (107) and 77.8% (375) were staged as N1 and N2. 78.2% (377) patients had postoperative recurrence.

The median follow-up for survivors was 26.0 months (IQR 22.8-29.2, range 2.0-125.0). During the follow up period, 364 out of 482 (75.5%) patients died. The median number of RML was 18.1% (range 1.8-100.0%) and the median number of NLN was 18 (range 0-86).

### Cut-off analysis to predict survival in NSCLC

**Figure 1:**
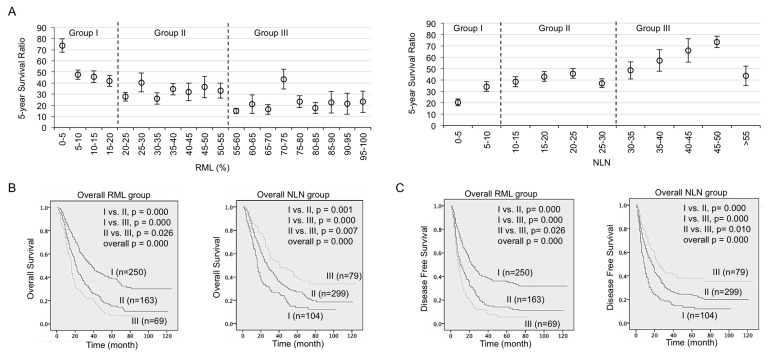
RML and NLN significantly predicted overall survival and disease free survival in NSCLC patients **A.** Scatterplot of 5 years survival ratio (mean and standard error) in patients grouped by RML at interval of 5% (left) and NLN at interval of 5 count (right). Optimal cut-off points were determined and used to stratify patients into 3 groups by each factor. **B.** Kaplan-Meier survival curves estimated the overall survival in RML groups (left) and NLN groups (right). **C.** Kaplan-Meier survival curves estimated the disease free survival in RML groups and NLN groups. Patient number in each group and *p* values in overall comparison and pairwise comparison were given.

Given that RML and NLN counts were continuous variable, cut-point analysis was performed to determine the cut-off counts that determine the greatest actuarial survival difference. Firstly, the RML (unit %) and NLN (unit count) variables were examined as categorical variables as shown in Figure 1A. As shown in Figure 1A left, 5 year survival ratio followed an increasing distribution and seemed were stable between subgroup #5 (RML 20.0-25.0) to subgroup #11 (RML 50.0-55.0). Therefore, patients were then stratified in to 3 subgroups (RML I, II and III) based on the RML counts 20.0 and 55.0, and survival was analyzed using the Kaplan-Meier method (Figure 1B left). Median overall survival was 38.0 months (95% confidence interval [CI], 31.0-45.0 months) for 250 patients in group RML I, 21 months (95% CI, 18.0-24.0 months) for 163 patients in group RML II and 16 months (95% CI, 14.0-18.0 months) for 69 patients in group RML III (all *p* < 0.001 in both overall comparisons and pairwise comparisons).

Similarly, NLN counts 10 and 30 were selected as the cut-off values and the patients were accordingly divided into the NLN I, II, III subgroups (Figure 1A right). Median overall survival was measured (38.0, 21.0 and 16.0 months for NLN I, II, III) and showed significantly difference (*p* < 0.001 in NLN I *vs*. II, NLN I *vs* III and *p* = 0.026 in NLN II *vs*. III) (Figure 1B right). Similar to the OS analysis, Kaplan survival analysis of DFS showed both NLN and RML are also significant predictors for DFS (Figure 1C).

**Figure 2:**
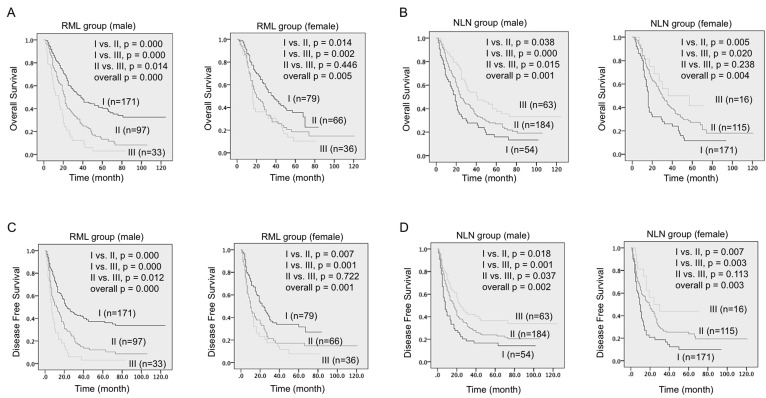
Survival differences among three RML and NLN groups were significant in male patients **A.** & **B.** Kaplan-Meier survival curves estimated the overall survival of male patients (A) and female patients (B) in three RML groups. **C.** & **D.** Kaplan-Meier survival curves estimated the disease free survival of male patients (C) and female patients (D) in three NLN groups. Patient number in each group and *p* values in overall comparison and pairwise comparison were given.

To explore the predict value of RML and NLN in each gender, we included gender as a stratum in Kaplan survival analyses (Figure 2). Both OS and DFS survival differences between each group (RML I-III and NLN I-III) and each stratum were calculated and compared. Notably, again this data suggested RML and NLN are strong risk predictors for male patients in both OS (left panels of Figure 2A and 2B) and DFS analysis (left panels of Figure 2C and 2D) in all comparisons. In contrast to that, there is one paired comparison (group II *vs*. III) in both female RML and NLN groups showed no significant survival difference in either OS (right panels of Figure 2A and 2B, *p* = 0.446 and 0.238) or DFS analysis (right panels of Figure 2C and 2D, *p* = 0.722 and 0.113).

Taking together, we concluded identification of useful cut-off values that stratify patients into different prognostic groups. Our results also indicated higher RML or lower NLN predicted worse outcomes of NSCLC patients. Moreover, our results also suggested RML and NLN were actually more accurate and powerful in predicting survival outcome for male patients compared to female patients.

### Univariate and multivariate survival analysis

As shown in Table 2, univariate analysis using the log-rank test were performed using 13 variables, including RML and NLN counts that coded as categorical variables with cut-offs identified in Figure 1A. Eight parameters were founded as statistically significant prognostic factors for OS: subcarinal lymph node (*p* < 0.001), T Stage (*p* = 0.023), N Stage (*p* < 0.001), pathological Stage (*p* < 0.001), Postoperative recurrence (*p* < 0.001), adjuvant chemotherapy (*p* = 0.011), as well as RML (*p* < 0.001) and NLN (*p* < 0.001).

**Table 2:** Univariate analysis of overall survival in all patients

Variable		5-YSR	χ^2^	*p* value
Gender	Male	25.2%	0.354	0.552
	Female	23.2%		
Age at surgery	<65y	24.4%	0.361	0.548
	>=65y	24.7%		
Smoking	Yes	24.5%	0.320	0.571
	No	24.4%		
Type of Pulmonary Lobectomy	Total	20.7%	5.328	0.070
	Lobe	25.0%		
	Sleeve	57.1%		
Location of Primary Tumor	Peripheral	24.5%	1.045	0.307
	Central	24.4%		
Subcarinal Lymph Node	Positive	31.6%	25.450	0.000**
	Negative	13.6%		
T Stage	T1	28.0%	7.559	0.023*
	T2	25.1%		
	T3	11.5%		
N Stage	N1	42.1%	22.329	0.000**
	N2	19.5%		
Pathological Stage	IIa	41.6%	21.296	0.000**
	IIb	43.5%		
	IIIa	19.9%		
Postoperative recurrence	Yes	9.8%	132.395	0.000**
	No	77.1%		
Adjuvant chemotherapy	Yes	26.6%	6.450	0.011*
	No	19.1%		
RML	I	36.0%	53.968	0.000**
	II	14.1%		
	III	7.2%		
NLN	I	13.5%	24.522	0.000**
	II	24.7%		
	III	38.0%		

RML, Metastatic Lymph Node Ratio; NLN, Negative Lymph Node; 5-YSR, 5-year survival rate. *, *p* < 0.05; **, *p* < 0.01.

Table 3 showed a multivariate analysis incorporating all the 8 potential important factors identified from the univariate survival analysis. There was strong evidence that, after controlling for other factors, only 3 variables (recurrence status, adjuvant chemotherapy and RML) were associated with OS of the patients and considered as independent predictors of survival. Patients with postoperative recurrence had worse OS than patients with no recurrence (hazard ratio [HR] = 0.121, 95% CI: 0.078-0.187, *P* < 0.001). In contrast to that, patients received adjuvant chemotherapy had better OS (HR = 1.614, 95% CI: 1.286-2.026, *P* < 0.001) and patients with lower RML had better OS (HR = 1301, 95% CI: 1.068-1.585, *P* < 0.01).

**Table 3:** Multivariate analysis of overall survival in all patients

Variable	HR	95% CI		*p* value
		Lower	Upper	
Subcarinal Lymph Node (+ *vs*. -)	1.135	0.885	1.456	0.318
T Stage (T1 *vs*. T2 *vs*. T3)	1.093	0.994	1.202	0.068
N Stage (N1 *vs*. N2)	1.041	0.474	2.283	0.921
Pathological Stage (IIa *vs*. IIb *vs*. IIIa)	1.060	0.682	1.648	0.795
Postoperative recurrence (+ *vs*. -)	0.121	0.078	0.187	0.000**
Adjuvant chemotherapy (+ *vs*. -)	1.614	1.286	2.026	0.000**
RML (I *vs*. II *vs*. III)	1.301	1.068	1.585	0.009**
NLN (I *vs*. II *vs*. III)	0.902	0.724	1.124	0.359

*, *p* < 0.05; **, *p* < 0.01.

### Combination of NLN and RML in predicting the OS

Although NLN was found not an independent predictor of survival, our correlation analysis showed NLN was highly correlated with two independent predictors, RML and recurrence status (Table 4 and Table S1). Next, we hypothesized NLN may add prognostic significance when incorporated with RML. First, we divided the patients into three RML groups and then three NLN subgroups within each RML group. A table including the 5-YSR analyses between NLN subgroups within individual RML groups was showed in Table S2. No significant overall difference was observed in each RML groups (all *p* > 0.05), which indicated this combination grouping did not work.

**Table 4:** Correlations between NLN with 3 independent OS predictors

	NLN	RML	Recurrence	Adjuvant chemotherapy
NLN (I *vs*. II *vs*. III)	1	-0.652**	0.091*	-0.005
RML (I *vs*. II *vs*. III)		1	-0.131**	-0.003
Postoperative recurrence (+ *vs*. -)			1	0.071
Adjuvant chemotherapy (+ *vs*. -)				1

Data given as the Pearson's correlation coefficient (R). *, *p* < 0.05; **, *p* < 0.01.

**Figure 3:**
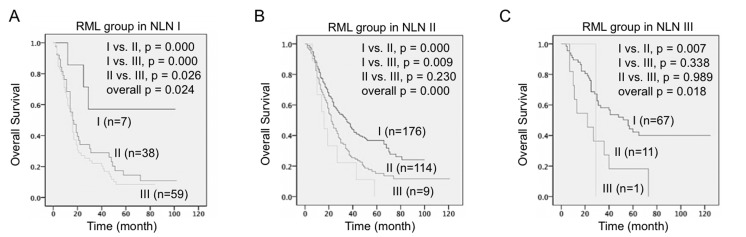
Combination of RML and NLN predicted overall survival in NSCLC patients Kaplan-Meier survival curves estimated the overall survival of NSCLC patients stratified by RML and NLN combination. **A.** Three RML subgroups in NLN I patients. **B.** Three RML subgroups in NLN II patients. **C.** Three RML subgroups in NLN III patients. Patient number in each group and *p* values in overall comparison and pairwise comparison were given.

Next we divided the patients into three NLN groups first and then three RML subgroups within each NLN group (Table 5 and Figure 3). Significant differences were found between the RML I-III in individual NLN groups and compared with the difference of RML I-III in overall population. Importantly, the 5-year survival rate (5-YSR) was much higher in RML I + NLN I, RML I +NLN III subgroups compared to the overall RML I subgroup (57.1% *vs*. 36.0%, 43.3% *vs*. 36%) and are much lower in RML III + NLN II and RML III + NLN III subgroups compared to the overall RML III subgroup (0% *vs*. 7.2%, 0% *vs*. 7.2%,). In conclusion, NLN is not only a strong prognostic factor for NSCLC patients, but also a useful marker for NSCLC classification when combined with RML.

**Table 5:** 5-YSR analyses between RML subgroups within each NLN group

	Overall		NLN I		NLN II		NLN III	
	n(N)	5-YSR	n(N)	5-YSR	n(N)	5-YSR	n(N)	5-YSR
RML I	90(250)	36.0%	4(7)	57.1%	57(176)	32.4%	29(67)	43.3%
RML II	23(163)	14.1%	5(38)	13.2%	17(114)	14.9%	1(11)	9.1%
RML III	5(69)	7.2%	5(59)	8.5%	0(9)	0.0%	0(1)	0.0%
I *vs*. II *p*	0.000**		0.000**		0.000**		0.007**	
I *vs*. III *p*	0.000**		0.000**		0.009**		0.338	
II *vs*. III *p*	0.026*		0.026*		0.230		0.989	
Overall *p*	0.000**		0.024*		0.000**		0.018*	

Date in each group was given as survived patient number (n), total patient number (N), and 5 Years Survival Ratio (5-YSR). *, *p* < 0.05; **, *p* < 0.01.

## DISCUSSION

Although significant attempts have been made to identify new molecular factors for NSCLC prediction [21-24], the lymph node tumor metastasis reflected by RML remains one of the fundamental prognostic factors that give prediction for survival status and guide for post-surgery therapy [10]. Disease management for NSCLC has recently evolved into a multidisciplinary setting and now requires more accurate disease staging by classifying patients into multiply prognostic subgroups with different clinical outcome. In addition to the current understanding of various important prognostic factors, our results showed that the prognostic value of RML for poor outcome in NSCLC patients with N1 and N2 disease was significantly enhanced by another prognostic factor NLN.

As described in 7th lung cancer TNM staging system, surgical-pathological staging such as pathologic T and N staging provide consistent classification guide in respect of the anatomic extent of tumor and is therefore a critical tool in lung cancer treatment and caring [25, 26]. Given that the prognostic value of the T and N staging is controversial due to the disputation of the definitions and complexity in clinical practices, many studies have tested the prognostic capacity of other predictive factors for OS in various types of cancer [27-29].

In clinical practice, NSCLC patients with resections are usually offered lymph nodes examination and postoperative adjuvant chemotherapy. Compared to the isolated number of metastatic lymph nodes, the positive ratio of metastatic lymph nodes is now widely accepted as a better prognostic indicator in cancer patients including N1 and N2 stage NSCLC (reported separately) and N3 stage gastric cancer [30-32]. In the presented study, both OS and DFS data from almost 500 NSCLC patients in stage II/IIIa were analyzed. Consistent to other reports, our data revealed RML was an independent prognostic factor highly correlated with OS and DFS (Table 2 and Table 3), which was used to stratify patients into three groups that should be considered when selecting treatment (Figure 1A). Interestingly, we also found RML and NLN predict survival differently in male and female patients (Figure 2). More specifically, RML and NLN were actually more accurate and powerful in predicting survival outcome for male patients. This suggested the gender preference should be considered when using RML and NLN to predict survival.

About 10 years ago, the number of tumor-free lymph nodes was firstly reported with potential prognostic significance in patients with lymph node-negative breast cancer [33, 34]. During the past few years, some impact studies began to take account of how NLN might be a useful tool in survival prediction, including in breast cancer [18], thoracic esophageal squamous cell cancer [15], late stage rectal cancer [35] and gastric cancer [36, 37]. For example, Wu et al reported that the NLN is associated with disease-free survival in patients with four or more positive lymph nodes after postmastectomy radiotherapy [38]. Since the limited number of NLN studies has never included the prognostic value of NLN in NSCLC patients, our data here report for the first time that NLN, in oppose to RML, is highly associated with both OS and DFS in NSCLC stage II/IIIa patients (Table 2) and can be used as a criteria to stratify the patients into separate groups with different outcome (Figure 1B and 1C). In consistent with the optimal cut-off points for NLN count reported in gastric cancer (NLN = 10, 15) [36] and breast cancer (NLN = 12) [38] we identified two useful cut-off points (NLN = 10, 30) in NSCLC. Another study of gastric cancer found the number of NLN is an independent prognostic factor which actually provide better accurate prognostic information when combined with N stage [37]. However, our multivariate analysis showed NLN is not an independent prognostic factor of NSCLC.

More and more studies showed NLN has an important prognostic impact when combined with other prognostic factors. Another example is NLN, again in combination with N stage, showed an improved prognostic value compared to number of removed lymph nodes, lymph node ratio, number of negative lymph nodes, and log odds of positive lymph nodes in breast cancer patients [14]. Interestingly, our correlation analysis suggested NLN, although was not an independent prognostic factor but strongly correlated with the independent factors RML and postoperative recurrence (Table 4). Compared to the recurrence status, apparently NLN showed stronger accuracy in stratifying the patients into different subgroups. Hence, we hypothesized the combination of NLN (cut-off points 10 and 30) and RML (cut-off points 20.0% and 55.0%) might achieve the best prognostic value for NSCLC patients. Indeed, our result showed low RML value indicated better survival in low NLN plus low RML group (NLN I + RML I, 5-YSR = 57.1%) while the survival benefit was completed lost in patients with high NLN plus high RML (RML III + NLN II/III, 5-YSR = 0 %) (Table 5). It is noted that high NLN plus low RML group (NLN III + RML I, total patient number = 67) also showed good survival (5-YSR = 43.3%), which probably is even more convictive than data of low RML plus low NLN group (total patient number = 7) due to the sample size difference. All together, we concluded, the addition of NLN classification (in prior to RML classification) allows identifying the NSCLC patients with RML <20% & NLN <10 may have the best postoperative outcome while the patients with RML <55% & NLN >10 may have the worst outcome.

To our best understanding, this study is the first study evaluated the important prognostic value of NLN and its enhancement to RML in accurate outcome prediction of node-positive NSCLC patients. The results of this study should be interpreted in the light of certain restrictions. For example, we cannot adjust in our analysis is the factors that causally affected NLN, including the extent of resection, number of nodes examined and the operation types. Despite these potential limitations, our study results firmly demonstrated a multiple factor model provides accurate predictions that inform NSCLC patients and allows for informed decisions to improve patient outcomes.

## SUPPLEMENTARY MATERIALS TABLES



## References

[1] Molina JR, Yang P, Cassivi SD, Schild SE, Adjei AA (2008). Non-small cell lung cancer: epidemiology, risk factors, treatment, and survivorship. Mayo Clin Proc.

[2] Osarogiagbon RU, Yu X (2013). Nonexamination of lymph nodes and survival after resection of non-small cell lung cancer. Ann Thorac Surg.

[3] Yang M, Cao H, Guo X, Zhang T, Hu P, Du J, Liu Q (2013). The number of resected lymph nodes (nLNs) combined with tumor size as a prognostic factor in patients with pathologic N0 and Nx non-small cell lung cancer. PLoS One.

[4] Osarogiagbon RU, Allen JW, Farooq A, Berry A, Spencer D, O’Brien T (2010). Outcome of surgical resection for pathologic N0 and Nx non-small cell lung cancer. J Thorac Oncol.

[5] Vielva LR, Jaen MW, Alcacer JA, Cardona MC (2014). State of the art in surgery for early stage NSCLC-does the number of resected lymph nodes matter?. Transl Lung Cancer Res.

[6] Shersher DD, Liptay MJ (2015). Status of sentinel lymph node mapping in non-small cell lung cancer. Cancer J.

[7] Bando E, Yonemura Y, Taniguchi K, Fushida S, Fujimura T, Miwa K (2002). Outcome of ratio of lymph node metastasis in gastric carcinoma. Ann Surg Oncol.

[8] Taylor MD, Lapar DJ, Thomas CJ, Persinger M, Stelow EB, Kozower BD, Lau CL, Jones DR (2013). Lymph node ratio predicts recurrence and survival after R0 resection for non-small cell lung cancer. Ann Thorac Surg.

[9] Li Q, Zhan P, Yuan D, Lv T, Krupnick AS, Passaro A, Brunelli A, Smeltzer MP, Osarogiagbon RU, Song Y (2016). Prognostic value of lymph node ratio in patients with pathological N1 non-small cell lung cancer: a systematic review with meta-analysis. Transl Lung Cancer Res.

[10] Sun G, Xue L, Wang M, Zhao X (2015). Lymph node ratio is a prognostic factor for non-small cell lung cancer. Oncotarget.

[11] Tamura M, Matsumoto I, Saito D, Yoshida S, Takata M, Takemura H (2016). Lymph node ratio as a prognostic factor in patients with pathological N2 non-small cell lung cancer. World J Surg Oncol.

[12] Port ER, Patil S, Stempel M, Morrow M, Cody HS (2010). Number of lymph nodes removed in sentinel lymph node-negative breast cancer patients is significantly related to patient age and tumor size: a new source of bias in morbidity assessment?. Cancer.

[13] Ma M, Tang P, Jiang H, Gong L, Duan X, Shang X, Yu Z Number of negative lymph nodes as a prognostic factor in esophageal squamous cell carcinoma. Asia Pac J Clin Oncol. 2016 Aug 4.

[14] Wu SG, Wang Y, Zhou J, Sun JY, Li FY, Lin HX, He ZY (2015). Number of negative lymph nodes should be considered for incorporation into staging for breast cancer. Am J Cancer Res.

[15] Zhu Z, Chen H, Yu W, Fu X, Xiang J, Li H, Zhang Y, Sun M, Wei Q, Zhao W, Zhao K (2014). Number of negative lymph nodes is associated with survival in thoracic esophageal squamous cell carcinoma patients undergoing three-field lymphadenectomy. Ann Surg Oncol.

[16] Deng J, Zhang R, Zhang L, Liu Y, Hao X, Liang H (2013). Negative node count improvement prognostic prediction of the seventh edition of the TNM classification for gastric cancer. PLoS One.

[17] Deng J, Sun D, Pan Y, Zhang L, Zhang R, Wang D, Hao X, Liang H (2012). Ratio between negative and positive lymph nodes is suitable for evaluation the prognosis of gastric cancer patients with positive node metastasis. PLoS One.

[18] Wu SG, Sun JY, Zhou J, Li FY, Lin Q, Lin HX, Guan XX, He ZY (2015). Number of negative lymph nodes is associated with disease-free survival in patients with breast cancer. BMC Cancer.

[19] Chen Y, Zhang L, Tian J, Ren X, Hao Q (2013). Combining the negative lymph nodes count with the ratio of positive and removed lymph nodes can better predict the postoperative survival in cervical cancer patients. Cancer Cell Int.

[20] Ke B, Song XN, Liu N, Zhang RP, Wang CL, Liang H (2014). Prognostic value of the lymph node ratio in stage III gastric cancer patients undergoing radical resection. PLoS One.

[21] Kilvaer TK, Paulsen EE, Hald SM, Wilsgaard T, Bremnes RM, Busund LT, Donnem T (2015). Lymphangiogenic Markers and Their Impact on Nodal Metastasis and Survival in Non-Small Cell Lung Cancer--A Structured Review with Meta-Analysis. PLoS One.

[22] Jiang L, Liang W, Shen J, Chen X, Shi X, He J, Yang C (2015). The impact of visceral pleural invasion in node-negative non-small cell lung cancer: a systematic review and meta-analysis. Chest.

[23] Wu H, Qi XW, Yan GN, Zhang QB, Xu C, Bian XW (2014). Is CD133 expression a prognostic biomarker of non-small-cell lung cancer? A systematic review and meta-analysis. PLoS One.

[24] Ren W, Mi D, Yang K, Cao N, Tian J, Li Z, Ma B (2013). The expression of hypoxia-inducible factor-1alpha and its clinical significance in lung cancer: a systematic review and meta-analysis. Swiss Med Wkly.

[25] Detterbeck FC, Chansky K, Groome P, Bolejack V, Crowley J, Shemanski L, Kennedy C, Krasnik M, Peake M, Rami-Porta R (2016). The IASLC Lung Cancer Staging Project: Methodology and Validation Used in the Development of Proposals for Revision of the Stage Classification of NSCLC in the Forthcoming (Eighth) Edition of the TNM Classification of Lung Cancer. J Thorac Oncol.

[26] Mirsadraee S, Oswal D, Alizadeh Y, Caulo A, van Beek E (2012). The 7th lung cancer TNM classification and staging system: Review of the changes and implications. World J Radiol.

[27] Veronese N, Nottegar A, Pea A, Solmi M, Stubbs B, Capelli P, Sergi G, Manzato E, Fassan M, Wood LD, Scarpa A, Luchini C (2016). Prognostic impact and implications of extracapsular lymph node involvement in colorectal cancer: a systematic review with meta-analysis. Ann Oncol.

[28] Paiella S, Sandini M, Gianotti L, Butturini G, Salvia R, Bassi C (2016). The prognostic impact of para-aortic lymph node metastasis in pancreatic cancer: A systematic review and meta-analysis. Eur J Surg Oncol.

[29] Li Y, Du P, Zhou Y, Cheng Q, Chen D, Wang D, Sun T, Zhou J, Patel R (2014). Lymph node micrometastases is a poor prognostic factor for patients in pN0 gastric cancer: a meta-analysis of observational studies. J Surg Res.

[30] Komatsu S, Ichikawa D, Miyamae M, Kosuga T, Okamoto K, Arita T, Konishi H, Morimura R, Murayama Y, Shiozaki A, Kuriu Y, Ikoma H, Nakanishi M (2016). Positive Lymph Node Ratio as an Indicator of Prognosis and Local Tumor Clearance in N3 Gastric Cancer. J Gastrointest Surg.

[31] Qiu C, Dong W, Su B, Liu Q, Du J (2013). The prognostic value of ratio-based lymph node staging in resected non-small-cell lung cancer. J Thorac Oncol.

[32] Yamashita K, Hosoda K, Ema A, Watanabe M (2016). Lymph node ratio as a novel and simple prognostic factor in advanced gastric cancer. Eur J Surg Oncol.

[33] Blancas I, Garcia-Puche JL, Bermejo B, Hanrahan EO, Monteagudo C, Martinez-Agullo A, Rouzier R, Hennessy BT, Valero V, Lluch A (2006). Low number of examined lymph nodes in node-negative breast cancer patients is an adverse prognostic factor. Ann Oncol.

[34] Salama JK, Heimann R, Lin F, Mehta N, Chmura SJ, Singh R, Kao J (2005). Does the number of lymph nodes examined in patients with lymph node-negative breast carcinoma have prognostic significance?. Cancer.

[35] Li Q, Zhuo C, Cai G, Li D, Liang L, Cai S (2014). Increased number of negative lymph nodes is associated with improved cancer specific survival in pathological IIIB and IIIC rectal cancer treated with preoperative radiotherapy. Oncotarget.

[36] Deng J, Liang H, Wang D, Sun D, Ding X, Pan Y, Liu X (2010). Enhancement the prediction of postoperative survival in gastric cancer by combining the negative lymph node count with ratio between positive and examined lymph nodes. Ann Surg Oncol.

[37] Shi RL, Chen Q, Ding JB, Yang Z, Pan G, Jiang D, Liu W (2016). Increased number of negative lymph nodes is associated with improved survival outcome in node positive gastric cancer following radical gastrectomy. Oncotarget.

[38] Wu SG, Sun JY, Zhou J, Li FY, Zhou H, Lin Q, Lin HX, Bao Y, He ZY (2014). Number of negative lymph nodes can predict survival of breast cancer patients with four or more positive lymph nodes after postmastectomy radiotherapy. Radiat Oncol.

