# Supplementary material for: Prognostic value of number of negative lymph node in patients with stage II and IIIa non-small cell lung cancer
Source: Oncotarget. 2017 May 24;8(45):79387–96. doi: 10.18632/oncotarget.18154 (PMC5668050; doi:10.18632/oncotarget.18154)
Supplement: Supplementary file 1 [file oncotarget-08-79387-s001.pdf]

Prognostic value of number of negative lymph node in patients with stage II and IIIa non-small cell lung cancer

Supplementary Material

Supplementary Table S1: Correlation between clinical variables with RML and NLN

Variables		RML groups (I, II, III)	NLN groups (I, II, III)
Gender (Male vs. Female)	Pearson's R	.148**	-.172**
	p value	.001	.000
Age (<65y vs. >=65y)	Pearson's R	-.141**	.093*
	p value	.002	.041
Smoking (Yes vs. No)	Pearson's R	.200**	-.200**
	p value	.000	.000
Pulmonary Lobectomy Type (Total vs. Lobe vs. Sleeve)	Pearson's R	.370**	-.224**
	p value	.000	.000
Tumor Location (Peripheral vs. Central)	Pearson's R	-.035	.056
	p value	.449	.221
Subcarinal Lymph Node (+ vs. -)	Pearson's R	-.050	.077
	p value	.269	.091
T Stage (T1 vs. T2 vs. T3)	Pearson's R	.439**	-.339**
	p value	.000	.000
N Stage (N1 vs. N2)	Pearson's R	.025	-.012
	p value	.583	.789
Pathological Stage (IIa vs. IIb vs. IIIa)	Pearson's R	.393**	-.257**
	p value	.000	.000
Postoperative recurrence (+ vs. -)	Pearson's R	-.122**	.094*
	p value	.007	.040
Adjuvant Chemotherapy (+ vs. -)	Pearson's R	-.006	-.022
	p value	.897	.628

*, $p<0.05$; **, $p<0.01$.

Supplementary Table S2: 5-YSR analyses between NLN subgroups within each RML group

	Overall		RML I		RML II		RML III	
	n(N)	5-YSR	n(N)	5-YSR	n(N)	5-YSR	n(N)	5-YSR
NLN I	14(104)	13.5%	4(7)	57.1%	5(38)	13.2%	5(59)	8.5%
NLN II	74(299)	24.7%	57(176)	32.4%	17(114)	14.9%	0(9)	0.0%
NLN III	30(79)	38.0%	29(67)	43.3%	1(11)	9.1%	0(1)	0.0%
Overall p	0.000**		0.084		0.822		0.953	

Date in each group was given as survived patient number (n), total patient number (N), and 5 Years Survival Ratio (5-YSR). **, $p<0.01$.
